# Integrating Natural Language Processing to Assess Effect of Loneliness and Social isolation on Cognitive Decline in Dementia

**DOI:** 10.1002/alz70861_108098

**Published:** 2025-12-23

**Authors:** James A C Myers, Nemanja Vaci

**Affiliations:** ^1^ University of Sheffield, Sheffield UK

## Abstract

**Background:**

Social isolation and loneliness are recognized as priority public health problems, showing impact on dementia development and progression.

**Method:**

Reports of social isolation, loneliness, and Montreal Cognitive Assessment (MoCA) scores were extracted from dementia patients' medical records using bespoke Natural Language Processing models and analyzed using mixed‐effects models.

**Result:**

Lonely patients (n = 382) showed lower MoCA scores throughout the disease (B = ‐0.83, t = ‐2.64, *p* = 0.008). Socially isolated patients (n = 523) experienced faster cognitive decline six months before diagnosis (B = ‐0.21, t = ‐2.18, *p* = 0.029), but were comparable to controls (n = 3912) before this period. This led to lower MoCA scores at diagnosis (B = ‐0.69, t = ‐2.53, *p* = 0.011) and in later stages.

**Conclusion:**

Lower cognitive levels in lonely and socially isolated patients suggest that these factors may contribute to dementia progression.

